# A concise guide to setting up a germ-free unit

**DOI:** 10.1016/j.xpro.2026.104709

**Published:** 2026-07-16

**Authors:** Romuald Binet, Claire F. Pearson

**Affiliations:** 1 Mary Lyon Centre at MRC Harwell, Didcot, Oxfordshire OX11 0RD, UK; 2 Kennedy Institute for Rheumatology, University of Oxford, Oxford, Oxfordshire OX3 7FY, UK

**Keywords:** Immunology, Microbiology, Model Organisms, NMGN Focused Collection

## Abstract

This overview provides novices with the most important factors to consider when planning to set up a germ-free facility to house sterile mice. This includes deciding what facility model is most appropriate for the required project, which will depend on the project length, size, space, and financial constraints. This guide addresses the most common facility models and the recommended workflows to maintain germ-free animals, providing a brief description of transport options. Information has been included regarding the possible ways of testing for germ-free status within a facility, as well as possible sterilants that can be used. The ideal germ-free facility set up is described, along withvarious options for facilities with greater constraints to allow for flexibility without compromising on the essential requirements of maintaining germ-free mice.

## Introduction

In a report from the Académie des Sciences in 1885 Louis Pasteur argued that he had “often talked about the interest in feeding a young animal with pure (artificially depleted of microbes) nutritive matter from birth […] with the assumption that life would be impossible under these conditions”.^1^ This declaration may have started the development of methods and devices to breed and study germ-free (GF) organisms. The first GF isolators were attributed to Nuttal and Thierfelder in 1897^2^ until the development of the first modern devices by James Arthur Reyniers in the middle of the twentieth century,^3^ followed by Trexler and Reynolds.^4^

Since then, there has been growing appreciation of the role that the microbiota plays in gut health, and in shaping the physiology and pathophysiology of both animals and humans.^5^ One way to study this is to remove microbes entirely from an organism and investigate how this affects a biological process of interest, and the techniques used to achieve this have been refined over the past several decades by many dedicated germ-free researchers.^6^^,^^7^^,^^8^ Although this approach is a relatively blunt tool with several caveats, it has nonetheless proved invaluable to understand the role that our resident microorganisms play in development, homeostasis and disease susceptibility.

A major strength of germ-free animals is the ability to reintroduce selected microbial species to create “gnotobiotic” animals, models in which the microbial composition is defined and other variables can be well controlled.^9^^,^^10^^,^^11^ These can be single strains, complex defined communities, or even faecal microbiota transplant (FMT) from other animals or humans that could “re-wild” the microbiome.^12^ This only works when starting out with truly germ-free animals, making rigorous sterility essential. GF research therefore requires specialised, costly equipment and controlled workflows.

In this primer we will introduce some of the basic concepts to consider when starting to work with germ free animals, focusing on the most commonly used species, mice. We will discuss the ideal set up and options whilst taking practicalities and cost into account to enable a flexible approach that remains rigorously sterile. Topics covered include some basic considerations for approaching a new germ-free facility, how the ideal facility would be set up, and how to ensure it stays sterile over long periods of time. For detailed germ free protocols we recommend consulting existing resources such as that by Vowles *et al*.^13^

In 2025, we collected information from eight research institutions in the United Kingdom and Ireland, including our own, with different GF capabilities. We have used the data collected to make suggestions about the considerations needed when designing a GF unit (Table 1).

**Table 1:** Data collected in a survey from eight germ-free facilities in the UK and Ireland

Category	Most used	Description	Alternatives	Examples of other available suppliers
Isolators	NKP-Isotec^a^	Flexible film isolators, single- or double-tiers	Bell Isolation systems^b^ and CBC flexible film isolators^c^, NKP-Isotec rigid isolators	–
Transfert port	Rapid transport port system	Multiple suppliers available	DPTE® Getinge^d^, NKP Solid Biohazard Port^a^, NKP Quadro Lock Port^a^, Bell hatch with swing door port^b^	BioPharma Dynamics CRTP^e^, CRL Solutions ERTP^f^, Sychem ABC Transfer® Rapid Alpha Transfer Port^g^
Bedding	ALPHA-dri^h^	pure cellulose fibre	Corncob, Aspen chips	Datesand^i^, SAFE^j^
Nesting	Various	-	ALPHA Nest®^k^, ALPHA-twist®^l^, Nestlets^m^, Paper Wool^n^	–
Diet	Various	Autoclavable or irradiated	Teklad T.2018S^o^, SDS RM1A^p^, RM3^q^, SAFE D113^r^, Labdiet 5010^s^	Research Diet Inc.^t^
Water	Autoclaved RO water	Often centrally supplied	Chlorinated, deionised, Baxter sterile water^u^, Irradiated water 50Gy^v^	–
Enrichment	Polycarbonate	preferred because autoclavable	Wooden chew block, cardboard tube, chew sticks	Bio-Serv^w^, Datesand^i^, LBS Biotech^x^, SAFE^y^, Tecniplast^z^
Gnotobiotic caging solution	Tecniplast ISOcages P^aa^	individually sealed cages, positive pressure	Allentown Sentry SPP^bb^	–
Shipment containers	NKP-Isotec GNOTO M1^cc^	Specialised cages for the transport of germ-free mice	Flexible GF shipper from Taconic^dd^ or CBC^ee^, rigid shipper from NKP-Isotec^cc^	–
Irradiation	Datesand^ff^	Enrichment, bedding, diet can be double-bagged and irradiated	Teklad^gg^	STERIS Applied Sterilization Technologies (AST)^hh^, Sterigenics^ii^, Scapa Healthcare^jj^, Swann-Morton^kk^, PolarSeal^ll^

a Gnotobiotic Flexible Film Isolators: ISO-Type 1, Type 2, Type 3, Type 4.

b Bell Isolation Systems - Products.

c CBC | Flexible Film Isolators | Gnotobiotic.

d DPTE®-XS Alpha Port - the original Rapid Transfer Port (RTP) - Getinge.

e Clean Rapid Transfer Ports (CRTP) - BioPharma Dynamics.

f RAPID TRANSFER PORTS (ERTP) - CRL Solutions.

g ABC Transfer® Rapid Alpha Transfer Port - Sychem.

h ALPHA-dri® - Shepherd Specialty Papers.

i Eco Pure Bedding | Datesand.

j Bedding.

k ALPHA Nest® - Shepherd Specialty Papers.

l ALPHA-twist® Certified - Shepherd Specialty Papers.

m Nestlets – Ancare.

n Paper Wool | Datesand.

o insights.envigo.com/hubfs/resources/data-sheets/2018s-datasheet-0915.pdf.

p sds-diets.com/sds-wAssets/docs/rodent/sds_rm1a-p-_ds.pdf.

q sds-diets.com/sds-wAssets/docs/rodent/sds_rm3-p-_ds.pdf.

r safe-lab.com/safe-wAssets/docs/product-data-sheets/diets/tds_safe_d113_en_2407_publish.pdf.

s 5010 - Laboratory Autoclavable Rodent Diet.

t Home - Research Diets, Inc..

u Baxter Sterile Water - 1 Litre Pour Bottle - The Vet Store.

v Services – LBS.

w Rodent Enrichment Devices: Bio-Serv.

x Environmental enrichment – LBS.

y Enrichment.

z AMOUSEMENT Enrichment Portfolio | Tecniplast – Mouse Enrichment Tools - Tecniplast UK.

aa Gnotobiology - Tecniplast UK.

bb Sentry SPP Mouse Sealed Positive Pressure IVC - Allentown.

cc Technical Extras For Isolators & Biocontainment By NKP Isotec.

dd Animal Shipping Products from Taconic Biosciences | Taconic Biosciences.

ee CBC | Germ-Free Shipper Sleeve | Safe Transportation.

ff Gamma Irradiation as an Effective Form of Sterilisation in Research Settings | Datesand.

gg inotiv.com/hubfs/resources/data-sheets/envigo-teklad-irradiated-standard-diets-us.pdf.

hh Gamma Irradiation Processing | STERIS AST.

ii Gamma Irradiation | Gamma Sterilization Services.

jj Scapa Healthcare Medical Product Sterilization and Validation.

kk Swann-Morton (Services) Limited.

ll Sterilization for Medical Devices | PolarSeal.

## Do you need your own unit?

The first consideration when setting up a germ-free (GF) unit is deciding whether it is really necessary at all. It is not trivial and very expensive, particularly the equipment required, which is summarised in Table 2. Breeding GF mice requires the purchase of positive pressure isolators that are essentially bubbles in which mice and everything they require can be kept completely sterile. They can now be built in rigid plastic or flexible vinyl film, but their principle has changed little over the years^14^: the air is HEPA filtered and positively pressured to the outside to minimise contamination risk and everything that goes in must move through a port system. Isolators require space and the operation of them requires significant investment in equipment and personnel. When strict SOPs are followed then isolators are the safest way to maintain sterility. Whilst experiments can take place within them, it’s important to note that due to open top cages and cross contamination within an isolator only one microbial status can be held in any individual isolator. When working with mice with multiple microbiota (e.g., GF and two or three FMT samples) a separate isolator is needed for each one.

**Table 2:** Approximate costs of equipment

	Local experimental unit (0-1 isolator)	Hybrid model (4-6 isolators)	The ideal GF unit (10-12 isolators)
	£100–200K	£200–550K	£1.5–3.5M
**Breeding equipment**			

Positive pressure isolators	£0–30K depending on the isolator size	£50–200K depending on the isolators size and number	£1-2M depending on the isolators size and number
Transfer/port system	£0–20K depending on the type of transfer solution	£20–100K depending on the type of transfer solution	£200K-1M depending on the type of transfer solution

**Sterilisation**			

Autoclave 160–300L	£30-60K depending on supplier and capacity	£30–60K depending on supplier and capacity	£60-120K for two autoclaves depending on supplier and capacity
Autoclavable drum for consumables + accessories	∼£3.5K	∼£3.5K	∼£7K for two sets
Compressor + atomiser	∼£3K	∼£3K	∼£3K
Ethylene oxide steriliser	∼£5K	∼£5K	∼£5K
Hydrogen peroxide vapour system	-	£10–30K depending on supplier and model	£10-30K depending on supplier and model

**Gnotobiotic cages**			

Complete rack (48 cages)	£40–50K depending on supplier	£80–100K for two racks depending on supplier	£120-150K for three racks depending on supplier
Accessories (dunk tank, seal checker, autoclave rack)	£5–10K depending on supplier	£10–20K for two sets depending on supplier	£10-20K for two sets depending on supplier
Class II BSC equipped with a port	£15–25K depending on supplier and model	£15–25K depending on supplier and model	£15-25K depending on supplier and model

Whilst isolators have been around for decades, a recent advance has been the “isolator-within-a-cage” system, or gnotobiotic cages, where each cage is hermetically sealed except when plugged into the rack, where it receives HEPA filtered air. This allows for multiple different microbial statuses on one rack. This reduces the space requirements and manipulations can be performed within a class II biological safety cabinet (BSC) or a laminar flow hood (depending on risk to user), making it much more viable for small-scale microbiota work.^15^ However, due to the need to open up cages within a cabinet the risk of contamination is much higher than within an isolator, although it is possible to keep mice GF for months with strict SOPs.^16^^,^^17^ The ways in which isolators and isolated cage systems can be used within a facility are outlined in the next section.

This specialised equipment is expensive. Moreover, due to the sterility requirements and the labour-intensive nature of the work we estimate the cost of GF work to be about ten times greater than that within a normal specific pathogen-free (SPF) facility. This is a significant investment and will only be feasible if there is sufficient work to keep the unit going. We created a checklist to assist the prospective gnotobiologist in deciding on the most suitable formula (Figure S1). Whether and how much to invest should depend on the requirements of the end users, and there is no single model that applies to all situations. Considerations include the numbers of mice required, the time frame over which they are needed, the strains used and the experimental protocols required. Not every laboratory or institute needs to invest in its own facility. Fortunately, multiple options exist to accommodate different needs and resources, ranging from the state-of-the-art GF breeding units to smaller experimental setups, including scientific collaboration. Each of these categories has its own advantages and limitations, which are broadly described in Table 3.

**Table 3:** Types of GF facilities available

Model	Pros	Cons
GF breeding facility	Full autonomy Allows complex projects	Expensive Requires space
Local experimental unit	Little commitment Good for short term projects	Need to purchase GF mice Not suitable for large projects Requires skills maintenance
National facility	Service fees only No need for space or equipment in local unit Reproducibility Large scale	Reduced flexibility in scheduling Risks associated with transport

### Setting up your own GF breeding unit

A local breeding unit represents the most comprehensive approach to germ-free research. Breeding GF mice in-house provides full autonomy and long-term control over animal supply and experimental timelines, making this model particularly suitable for larger institutions that require a sustained and predictable availability of animals. However, this approach comes with substantial costs associated with specialist equipment, including GF isolators, autoclaves, and gnotobiotic cages. Such equipment requires significant space and must be operated under strict high-barrier conditions, with controlled access measures (e.g., limited entry, showering and dedicated clothing) to protect the colony. In addition, a GF breeding unit relies on highly trained personnel who are able to work under aseptic conditions and are proficient in sterilisation, transfer, and screening procedures. While this model offers the highest control and capacity, it also represents the highest level of financial and operational commitment and generally requires at least one “super-user” who can commit resources and substantial experimental usage over a number of years.

### Building a local experimental unit

For smaller groups, it may be more efficient to design a local experimental GF unit that lacks breeding capabilities and purchase and import experimental animals when required. This is especially useful for units where GF work is sporadic. The recently developed gnotobiotic cages provide an alternative to traditional GF isolators with a much smaller physical footprint. Gnotobiotic cages represent a huge advance due to their cage-level containment which allows the study of multiple microbiological conditions on a single rack with a smaller footprint than one isolator. On the other hand, handling these cages requires a high level of rigour to maintain sterility. They must be manipulated within a BSC and involve multiple sterilisation steps. In the absence of in-house breeding, animal supply is dependent on external availability, and GF mice are costly; however, access to a wide range of validated models can be a significant advantage for experimental research.

### The hybrid model

Some institutions may opt for a hybrid model that combines small-scale breeding with experimental capabilities. In this format, only essential and heavily used mouse models are bred locally, reducing dependence on external suppliers, while other strains are purchased as required. These facilities often also use gnotobiotic cages for experimental studies. Typically, this approach requires a limited number of isolators (for example, three to five), autoclaves, and core research equipment, including at least one BSC. The hybrid model offers flexibility while limiting the costs associated with a large breeding unit. It is particularly well suited to research groups that focus extensively on one or two mouse models and require a sustained supply, but do not wish to maintain multiple low-use strains. Nevertheless, it still demands dedicated space, a substantial initial investment, and trained personnel capable of working across both systems.

### The national facility option

To help cope with financial pressures, smaller groups may pool their resources and invest in a centralised or national germ-free facility. A large communal GF unit not only absorbs the cost of specialised infrastructure, equipment, and personnel, but also aligns well with the principles of the 3Rs—Replacement, Reduction, and Refinement. Managing multiple projects within a single specialised facility reduces the need for repeated breeding strategies, thereby lowering the overall number of animals used in research. This model also shifts responsibility for sterility assurance and animal welfare to highly qualified and recognised professionals. Centralised facilities are able to invest in advanced equipment and standardised protocols, which translate into improved reproducibility and reliability. The main drawbacks of this approach are reduced flexibility in experimental planning and the risks associated with transporting GF animals. However, in an era of tightening research budgets and increasing technical complexity, these limitations are often outweighed by the financial predictability and high level of consistency and robustness that a national GF facility can offer.

### Shipping

If not breeding all required strains in-house, or when importing a new strain from elsewhere, a straightforward transport system is required. Various methods for shipping GF mice between facilities have been developed, with the most popular being the GF shipper (such as from Taconic Biosciences or CBC) and the newer GF cage (such as from NKP). The shipper allows for the transport of up to three cages that are visible to inspectors on the journey without breaking sterility, and it can be directly linked to many isolator ports, allowing import and export of multiple mice. This is a good way to move breeding mice although in our experience the shipper can be cumbersome to link to the isolator port, it is very expensive and can be difficult to re-use due to sterility issues. The alternative is a GF cage that provides a filter top lid that can be temporarily sealed to allow full disinfection of every surface of the cage during importing. This can be autoclaved into an isolator in advance of shipping and is a much easier way to move mice between units, as well as being reusable. However, it can be complex to import live mice into an isolator as the sealing of the cage for disinfection only allows for limited air within the enclosure, meaning importation must be performed rapidly, which is often at odds with sterility protocols for isolators. In our experience, when shipping GF mice locally (e.g., within a day) or animals that come from or will go into multiple different isolators, the GF cage works well. When shipping for longer distances or several mice from within one isolator, the GF shipper may provide a more secure transport option. Nevertheless, good communication between the shipper and recipient well before a required shipment is organised, to ensure that the shipping method chosen is compatible with both units.

## How to design a GF unit

**Figure 1:**
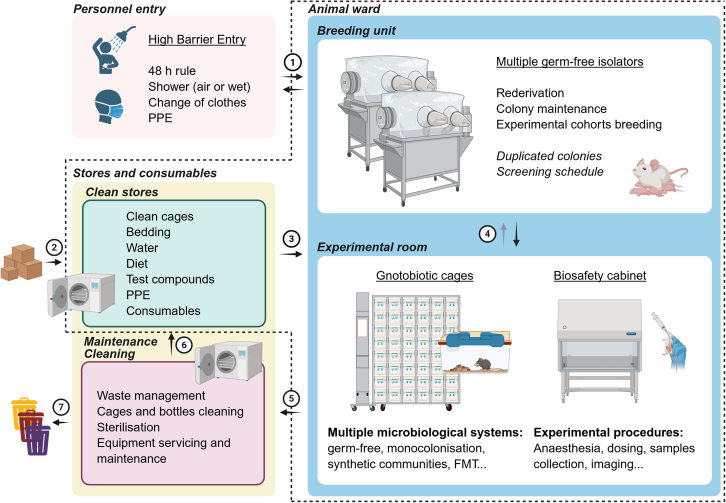
Overview of an ideal GF facility The dotted line represents the barrier between the clean inside area where animals are kept and the dirty outside area. (1) Personnel entry requires a shower (air or wet), a full change of clothes into PPE and all should follow a strict 48 h rule if they have visited another rodent facility, (2) Cages, consumables, equipment must be cleaned and decontaminated before entering the barrier, and (3) sterilised before moving to the animal ward. (4) The breeding section and the experimental section are physically separated and while movement between the two is possible, it is not recommended. (5) Waste and dirty cages or equipment are extracted from the clean area and processed in a service area where they can be cleaned and sterilised to be reused (6) or exported as waste (7). Created in BioRender. Binet, R. (2026) https://BioRender.com/3g86hso.

Whatever the eventual model chosen, a GF unit is a unique environment where the highest standards of animal welfare, consistent aseptic methods, and high-level experimental outputs coexist (Figure 1). At a minimum, it relies on (i) a unidirectional clean/dirty workflow, (ii) high-barrier processes that protect the animals’ GF status, (iii) validated sterilization methods, and (iv) a robust monitoring schedule. Drawing on our 2025 United Kingdom and Ireland survey—and acknowledging that there is no one-size-fits-all design, as discussed previously—the following sections describe what we consider the ideal configuration while offering practical alternatives to accommodate different budgets, space constraints, and research priorities.

### The ideal GF unit (unlimited budget and space)

The optimal GF facility segregates people, animals, and materials/consumables into dedicated zones with distinct barrier measures, summarised in Figure 1. Larger rooms allow for more flexibility – 50-70m^2^ is ideal to house 6-12 isolators (depending on isolator style). At least two staff members are required for operation of the facility at all times, but due to the physical requirements more personnel would increase flexibility of the unit. Personnel access via a changing room, take a shower, and don a clean change of clothes. Particle-based cleanliness can be kept through a mild positive pressure within the room and donning of new clothes and overshoes.^18^ Showering will not remove all microbes from staff but may reduce the bacterial burden briefly and has proved to be an effective way of physically limiting access to the facility, If the facility is under positive pressure and the air is HEPA filtered, this may help to keep the facility as clean as possible, thereby reducing contamination risk overall. Air showers may be an alternative that allow higher throughput entry. These measures are sufficient for routine isolator-based husbandry. When working with open gnotobiotic cages in a BSC, additional personal protective equipment (PPE)—for example, sterile oversleeves or gown and sterile gloves—is required to avoid contamination.

GF animals tend to be imported live; in that case, although testing records should be checked to ensure sterility, they must always be kept in quarantine upon arrival until their GF and health status are confirmed. Ideally new imports are housed in a completely separate isolator as this has the lowest risk of contaminating other strains or becoming contaminated. Alternatively, they could be kept within a gnotobiotic cage system, but this does risk contamination during the quarantine period if opened. Animals should be left for one to two weeks in quarantine before testing occurs, to allow time for any contaminants that arose during the transport process to build to a testable level. Allowing for testing time, it is therefore necessary to consider a potential overall quarantine period of around four weeks, so with limited isolators/cage systems, this requires careful planning. In addition to importation, mice may also be rederived from a specific pathogen-free (SPF) strain available within the institution through embryo transfer or hysterectomy.^13^ In this case it’s equally important to keep the animals quarantined and to perform rigorous testing for GF status before the rederivation can be considered as successful.

In an ideal setting all consumables and materials would be stored outside the barrier and sterilized before entering the facility, either by a gaseous method or by autoclaving. Where this is not possible storage within a separate room in the facility can also work well. It is important not to have too high a density of stored materials to ensure that regular cleaning within the room is possible to prevent the build-up of dust or potential spores on the outside packaging, as this will complicate the ability to decontaminate the items for importation. When required, materials are transferred to isolators using transfer sleeves or bags connected to port doors, or via a rapid-transfer system such as DPTE® Beta containers.

Opening an isolator to import or export supplies risks contamination. For flexible film isolators there is also the risk of tearing of the canopy itself, or rips in gloves that have degraded over time. To minimise the risk of such physical breaks in sterility each isolator should be checked for tears and holes daily, and each glove should be inspected every time it is used. Isolators are under positive pressure so an interruption to electrical supply could also compromise an isolator. To limit the risk of losing a colony due to contamination or other issues, colonies should ideally be duplicated across two completely independent isolators. It is important to consider genetic drift and the risk of highly inbred colonies so animals can be transferred between independent isolators to avoid the risk of colony divergence. Ideally such a transfer would occur through an intermediate (e.g., a transfer isolator) to avoid directly connecting the two isolators in case a contamination is present that might spread. While dimensions vary widely, a typical isolator can hold up to 20–30 cages while preserving space for a stock of consumables. It is important to ensure sufficient consumables are within the isolator to minimise the need to open it, so in a smaller isolator it can be better to have fewer cages in order to allow more space for consumables. In an ideal configuration that supports multiple breeding lines and concurrent projects, we estimate that a minimum of ten isolators is usually required to meet breeding needs.

GF isolators—most commonly flexible-canopy models—are designed to optimize operator access to cages and supplies, and it is generally possible to perform routine procedures such as weighing and oral dosing inside the canopy. For experimental work, however, especially when recolonising mice, it is recommended to transfer mice to gnotobiotic cages (ISOcage P from Tecniplast or Sentry SPP from Allentown) and implement aseptic protocols in a BSC. For more technical procedures—such as injections, sample collection, or surgery—the operator can open a gnotobiotic cage within a sterile Class II BSC while preserving a germ-free environment. The risk of contamination reduces when adding back microbes to animals, particularly more complex microbiotas, as these provide more colonization resistance to contaminants. Depending on the number of projects running concurrently, the facility may require several gnotobiotic racks and at least two BSCs.

All equipment must be regularly tested and serviced to prevent loss of integrity that could compromise GF status. In parallel, a robust monitoring program is essential to verify that the animals and their environment remain germ free throughout the initial breeding steps and across the experimental phase.

### A smaller but self-reliant GF unit (focused on a few animal models)

**Figure 2:**
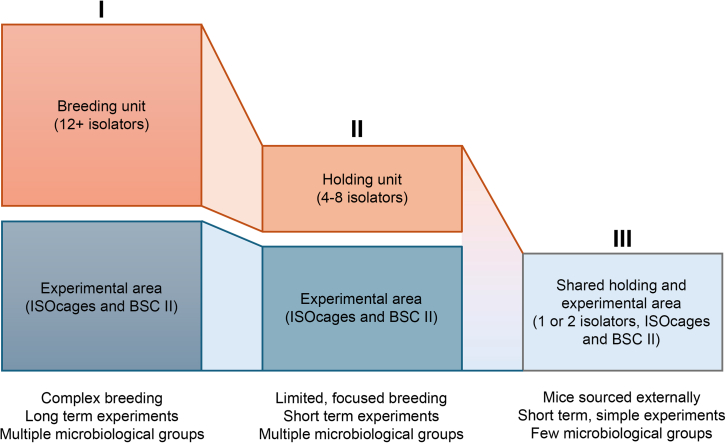
The different GF facilities approaches The scale, while representative, is only indicative of the relative size of each type of facility.

For institutions with moderate resources and a steady but not overwhelming demand for GF work, a hybrid model combining a small breeding unit with an experimental arm provides flexibility without the footprint of a full-scale facility (Figure 2). It may be essential for instance to maintain a colony of the animal model this group is primarily interested in rather than constantly relying on an external supplier, particularly if the model of interest is not commercially available.

Personnel, consumables and animal segregation remain the same and the need for PPE and high barrier measures is not affected by the scale. If inclusion of showers or air showers is not practical, strict exclusion criteria (e.g., no rodent contact for 48 hours previous to entering), gloves, masks, hair coverings and overshoes are even more critical protective measures that should be followed. In this tier, housing might consist of four to eight isolators used to maintain essential strains, complemented by one to two gnotobiotic cages racks that support short-term experiments and allow multiple cohorts to be run in parallel. Because of the reduced resources (smaller rooms, lower budget) the available equipment may be limited and shared between the breeding and the experimental sections. Sterilization usually relies on only one or two autoclaves for drums and cages, alongside validated chemical sterilants; irradiation of consumables is usually outsourced to external providers who have the appropriate validated equipment (Table 4). Gamma irradiation is usually performed to 50kGy for germ free consumables: most companies will provide a range of this rather than guarantee 50kGy across every part of every package. Because of this it’s advisable to keep consumables packages reasonably small and e.g., irradiate no more than 2kg of food within one bag, to maximise the radiation dose received by every single pellet. Transfer systems still use sleeves and drums, but the number of ports and the diversity of transfer systems are reduced compared to the ideal unit. Workflow is more compact, with shared sterilization and procedure areas serving both breeding and experimental activities, and strict SOPs are needed to prevent cross-contamination between zones and housing types.

**Table 4:** UK providers of gamma ionizing radiation from cobalt-60

Company	Dose available	Products irradiated	Link
Datesand	25–50 kGy	bedding, nesting, enrichment, edible goods, nestpaks, PPE, janitorial goods, small equipment, client’s products	scanbur.com/Files/Images/PDF/Datesand Product Guide_Issue 4.pdf
Teklad	20–50 kGy	bedding, enrichment, diet	Diet FAQs
STERIS	n/s	single-use medical devices, human, animal and synthetic tissue and pharmaceuticals	Gamma Irradiation Processing | STERIS AST
Sterigenics	up to 100 kGy	Animal Food & Ingredients, single-use medical products, packaged products, food products & ingredients, cosmetics, tissue-based devices, implantable medical devices (stents, heart valves, orthopedics), pharmaceutical products and packaging, combination medical devices that may contain a pharmaceutical or biologic, polymers, raw materials	Gamma Irradiation | Gamma Sterilization Services
Scapa Healthcare	15–55 kGy	Single-use medical products, pharmaceutical products and packaging, packaged products, food products, cosmetics and toiletries, tissue-based devices, implantable medical devices, combination medical devices and pharmaceutical or biologic products, raw materials	Scapa Healthcare Medical Product Sterilization and Validation
Swann-Morton	*n/s*	n/s	Swann-Morton (Services) Limited
PolarSeal	*n/s*	Medical devices	Sterilization for Medical Devices | PolarSeal

n/s not specified.

In practice, the capacity of such a unit ranges from approximately 300 to 600 mice, offering a balance between colony continuity and experimental throughput. This configuration reduces reliance on external suppliers while avoiding the complexity and cost of a large breeding unit, and it represents a realistic aspiration for many medium-sized institutions.

### The minimalist GF room (occasional studies)

For occasional GF studies, an experimental-only unit can deliver meaningful results with minimal infrastructure, provided that workflows are carefully designed and rigorously implemented. A laboratory that becomes interested in the role of the microbiome in their disease model may consider a limited investment before scaling up to a larger unit. One gnotobiotic cage rack next to a dedicated BSC II would be enough to perform simple, short-term experiments if strict SOPs and sterile conditions are respected.

Housing in this scenario consists of gnotobiotic cage racks or, in some cases, one or two small isolators used for short-term cohorts rather than long-term colony maintenance. A dedicated room may not be available and GF or gnotobiotic mice may share a ward with SPF mice on a regular IVC rack. Sterilization often relies on the already existing autoclave or on outsourced irradiation for diet, bedding, and cages, supplemented by chemical sterilants for ports and biosafety cabinet workflows. The workflow centres on the BSC II for all cage handling, with strict maintenance of clean and dirty zones and clear role allocation during critical tasks.

Capacity typically ranges from 100 to 300 mice, depending on rack size and study duration, and may fluctuate with the availability of GF animals from external suppliers. Although this lean setup operates at a fraction of the cost of a breeding unit, it demands meticulous planning, robust documentation, and well-trained staff to maintain sterility. This can be problematic when staff only occasionally perform these techniques, so it is necessary to perform regular refresher training, e.g., every three months to ensure that skills are not lost. When these conditions are met, it provides an agile and scientifically robust option for institutions that do not wish to operate a full GF colony.

## The sterility problem

Once you have the ideal GF facility set up for the required needs, the most important step to maintain GF status is to ensure that animals can be shown to be sterile. Confidence in GF status relies on being able to detect the presence of living microorganisms, which means the definition of being “GF” is only as good as the tests used – if only testing for contamination under certain conditions and there is something that grows in alternative conditions including within a live mouse, the animals will pass their “GF” test but won’t necessarily be completely sterile. Traditional GF testing focuses on checking for bacterial contamination, but it is important to consider the role of viruses and fungi as well and ensure that tests for different microorganisms are built into any testing regime, one may include for example an annual screen for infectious agents e.g., as recommended by FELASA.^19^ Most more frequent GF testing regimens focus on bacterial contamination. Due to the variety of microorganisms that exist no one test alone is sufficient to have confidence in GF status, so a number of different tests need to be performed.^20^^,^^21^ These can include culturing in aerobic and anaerobic conditions, PCR-based 16S analysis to amplify bacterial DNA and bacterial staining.^22^ These are outlined in Table 5 with detailed protocols published elsewhere.^23^^,^^24^ Testing can be performed either in-house or through external companies as several now offer GF monitoring services. Routine in-house monitoring provides flexibility and a rapid answer with access to primary data allowing a close assessment of borderline results, but also requires investment in staff training and time, as well as access to a microbiological safety cabinet, aerobic and anaerobic incubators. Staff training is essential to minimise the risk of accidental contamination and false positives that require repeated testing. Although slower to provide results, external monitoring does not require such specialist equipment and personnel investment but is more costly, and in order to perform anaerobic culturing externally, specialist tubes that can support anaerobic bacteria during transport are required. Using a combination of both systems where possible is ideal for speed, accuracy and cross-validation.

**Table 5:** Common methods of sterility testing for GF units

Method	Advantages	Disadvantages
Aerobic culture at 37°C	Clearest evidence of contamination	Some microbial species are slow-growing (1 to 2 weeks)
Anaerobic culture at 37°C	Clearest evidence of contamination	Some microbial species are slow-growing (1 to 2 weeks) If samples process rapidly, then nothing will grow Requires access to an anaerobic chamber
Aerobic culture at 23°C	Allows growth of agents that would not otherwise be picked up (including some fungi)	Some microbial species are slow-growing (1-2 weeks)
16S PCR	Rapid, highly sensitive May be possible to identify contaminants	May pick up presence of dead bacterial debris Can be more costly and requires more training Will detect bacteria species only, no fungi nor parasites
Gram staining	Rapid, cheap	Will not distinguish live from dead bacteria Can be difficult to interpret in faecal/caecal contents with food antigens present
Bacterial viability testing	Rapid, can detect live bacteria	Requires a fluorescent microscope Requires more training

### Screening

Swabs of the isolator as well as faecal/caecal samples from animals should be tested. These samples should be kept moist and dealt with rapidly to prevent death of sensitive microbes. Food, water and bedding should also be tested on a regular basis. We would recommend a minimum of culturing faecal/caecal contents at 37^o^C aerobically and anaerobically and at least one other method (such as PCR-based or bacterial staining), but ideally all three methods would be utilised. We would also recommend aerobic culture at room temperature to pick up environmental contaminants, as these can be the most common microorganism infections within an isolator. Any positive test result should be repeated to ensure it wasn’t a cross-contamination during sample preparation. Isolator swabs should be cultured aerobically and anaerobically. Each isolator should be tested on a regular basis (at least every two months) and more frequently for heavily populated isolators. It’s also important to be able to distinguish between the presence of dead microbes or parts of microbes (such as short sequences of DNA that may be amplified by PCR methods) and those organisms that remain viable and able to infect, hence the need for multiple techniques.

GF testing can also be extended to the environment within the isolator itself. Mould traps may be used to test imported food by grinding pellets, soaking them in water, and leaving them to incubate. If spores are present, visible mould may develop over time, indicating contamination and necessitating the rapid shutdown and decontamination of the isolator. However, some argue that mould traps may create unnecessary issues by allowing potentially harmless spores to grow, and the field remains divided on whether mould traps are beneficial overall.

### Sterilization of diet and consumables

Cages, enrichment, bedding, diet and water must be sterilised before entry in an isolator. Animal feed in particular poses the greatest risk of containing fungal spores and other microorganisms that survive the sterilisation process. Whilst most SPF facilities use pelleted diets, it is often preferable to use an extruded diet in GF conditions, as this is less dense^13^ allowing better penetration of sterilants. Because food typically carries the highest microbial load, it is essential that sterilisation procedures are sufficiently robust to eliminate all microbes, including spores. Spores are notoriously resistant to standard sterilisation methods such as steam autoclaving at 121^o^C and 15 psi for 30 minutes or conventional 25 kGy irradiation. Therefore, higher-intensity methods such as autoclaving at 132^o^C-134^o^C for 20-30mins (summarised in Figure S2) or irradiation at 50 kGy^25^ are required. A list of UK providers of gamma irradiation services is provided in Table 4.

To ensure that every pellet is fully sterilised, small-batch processing is recommended. This can be achieved by packaging feed into small bags or spreading pellets on trays within a sealed autoclavable drum, ensuring the layer is no more than one pellet thick. The same principles should be applied to the sterilisation of all items entering the GF environment (with the exception of water, which can only be autoclaved at 121^o^C for 20 minutes due to steam production). High temperature autoclaving can harden pellets, so it is crucial to choose a diet formulation that can withstand the process. Some nutrient loss will occur with either autoclaving^26^ or to a lesser extent with irradiation,^27^ particularly of vitamins such as thiamine,^28^^,^^29^ and previous studies are summarised in Tables 6 and 7. A high nutrient diet, ideally supplemented with vitamin K which is normally provided by intestinal bacteria,^30^ should therefore be used. Since feed must be formulated and processed to allow sufficient nutrients and other components, it is important to consider food choice in combination with sterilisation method, and they directly influence one another. If using a feed that hasn’t previously been validated for the sterilisation method used it is advisable to test the final nutrient composition to ensure that it meets the nutritional requirements of the animals.

**Table 6:** Heat and radiation effects on vitamins commonly added to lab rodent diets

Vitamin name	Alternative name	Tuśnio et al.^a^	Zimmermann and Wostmann^b^	Reyniers et al.^c^
		1× 20′ 121°C or 1× 10′ 134°C steam	3× 25′ 123°C steam	1× 30′ 123°C steam
**Fat soluble vitamins**				

Vitamin A	Vitamin A Acetate, Retinol	Most affected by autoclaving	Lose 15-20%	−20%
Vitamin D3	Cholecalciferol	Most affected by autoclaving (D2)	−	−
Vitamin E	DL-Alpha Tocopheryl Acetate	not really changed	−	Not affected
Vitamin K3	Menadione Dimethylpyrimidinol Bisulfite	not really changed	−	−100%

**Water soluble vitamins**				−

Vitamin B1	Thiamine Mononitrate	not really changed	Lose up to 90%	−85%
Vitamin B2	Riboflavin-5-Phosphate	increased	Lose 5-10%	−40%
Vitamin B3	Nicotinic Acid (Niacin)	–	−	−15%
Vitamin B5	Calcium Pantothenate	Most affected by autoclaving	Lose up to 50%	−30%
Vitamin B6	Pyridoxine Hydrochloride	increased	Lose 10−30%	−20%
Biotin	Biotin	Not detected	−	Not affected
Vitamin B9	Folic Acid	Not detected	−	−60%
Vitamin B12	Vitamin B12 Supplement	–	−	−
Choline	Choline Chloride	–	−	−10%

a https://doi.org/10.22358/jafs/65672/2014.

b https://doi.org/10.1093/jn/79.3.318.

c https://doi.org/10.1093/jn/41.1.31.

**Table 7:** Vitamins content in regular vs autoclavable diets from two common suppliers

	Teklad regular (2018)	Teklad autoclavable (2018S)	SDS regular (RM1)	SDS autoclavable (RM1A)
**Fat soluble vitamins**				

Vitamin A (IU/g)	15	30	8554	45000
Vitamin D3 (IU/g)	1.5	2	621	4000
Vitamin E (IU/g)	110	135	84	200
Vitamin K3 (mg/kg)	50	100	10.1	60

**Water soluble vitamins**				

Vitamin B1 (mg/kg)	17	117	8.6	50
Vitamin B2 (mg/kg)	15	27	4.3	30
Vitamin B3 (mg/kg)	70	115	71.1	150
Vitamin B5 (mg/kg)	33	140	20.1	56.2
Vitamin B6 (mg/kg)	18	26	4.8	45
Vitamin B9 (mg/kg)	4	9	0.8	10
Vitamin B12 (mg/kg)	0.08	0.15	0.007	0.069
Biotin (mg/kg)	0.4	0.9	0.28	0.4
Choline (mg/kg)	1200	1200	1080	1500

1 IU vitamin A = 0.3 μg retinol.

1 IU vitamin D = 25 ng cholecalciferol.

1 IU vitamin E (natural form) = 0.67 mg alpha-tocopherol. 1 IU vitamin E (synthetic form) = 0.45 mg alpha-tocopherol.

The decision to autoclave or irradiate diet and some other supplies often comes down to practical factors such as access to specialised autoclave cycles or the cost and availability of irradiation services. Both methods are effective when performed correctly, but it is worth noting that, in our experience, switching between autoclaved and irradiated diets may alter *in vivo* phenotypes, possibly due to differing levels of residual antigens. Once a system is established and validated, maintaining consistency is strongly recommended.

When autoclaving it is essential to include indicators to ensure that the cycle has completed correctly before importing items into a GF environment. Ideally, both a biological and chemical indicator should be used and checked before importation, which requires placing them in locations visible from outside. Best practice also includes placing additional indicators in the area most difficult for steam to penetrate (such as the centre of a bag of food). These can be checked afterwards, and although sterility may already be compromised at that point, rapid action can still prevent further spread. At a minimum, when setting up autoclave cycles, extensive testing should be performed using indicators placed within the types of loads that will be processed. Ideally, these validation checks should be repeated every few months to confirm that the cycle still functions correctly.

### Isolator imports and exports

Sterilisation of products is only the first step. Sterile items must then be imported into the isolator. Whether autoclaving or irradiating, the basic procedure involves connecting the isolator port to the sealed port bag or autoclave drum containing the items, sterilising the connection, and finally breaking the seal to transfer the items inside.^24^ DPTE® port systems are an exception, as the beta-container attaches directly to the alpha-port on the isolator, removing the need for an additional connecting sleeve.

For all systems except (except DPTE®), the use of an air compressor is recommended to aerosolize the disinfectant. Ideally, an ultra-quiet compressor should be used to minimise disturbance to both personnel and animals. When using acid-based sterilants, stainless steel components should be used wherever possible to avoid chemical degradation. Selection of a sterilant depends on several factors, the most important being its ability to kill spores and other hard-to-kill species. Harsh chemicals capable of this will inevitably degrade equipment over time. The most common sterilants are chlorine dioxide, peracetic acid and hydrogen peroxide/ethylene oxide-based systems, and the advantages and disadvantages of each are outlined in Table 8. No sterilant is perfect, and some may have country-specific regulatory restrictions. It is essential to ensure staff safety during sterilisation procedures. This may require chemical-resistant gloves, sleeves, aprons and portable respirators, all of which add to the cost and complexity of running a GF unit.

**Table 8:** Common disinfectants and sterilants used in GF units

Sterilant	Method	Usage
Peracetic acid	Peracetic acid (PAA)	• Corrosive • Requires neutralisation before disposal • Acrid odour
TecCare Ultra^a^	Silver-stabilised PAA	• Corrosive • Requires neutralisation before disposal
Vapourised hydrogen peroxide (VHP)	Hydrogen peroxide (H2O2)	• Residue free • Requires expensive equipment • Requires venting
Endosan3^b^	Silver-stabilised H2O2	• Less efficient against spores • Low toxicity • pH neutral • May require longer sterilisation time
Spor-Klenz^c^	Combination: H2O2 + PAA	• Lower volatility compared to other PAA
Ethylene oxide	Ethylene oxide	• Carcinogen, mutagen, highly toxic • Requires aeration post-sterilisation
Clidox-S^d^	Chlorine dioxide	• Corrosive • Requires neutralisation before disposal • Not widely available or approved for use in the EU
MB-10^e^	Chlorine dioxide	• Less corrosive than standard chlorine dioxide formulations -concentration dependent • Available in tablets
Virkon^f^	Peroxygen-based	• Sporicidal only at higher concentrations and exposure time • Less corrosive than PAA (material compatibility dependent) • Stable in powder form and available in tablets • Activity reduced by organic matter

a TECcare ULTRA - Teccare.

b EndoSan Stabilised Hydrogen Peroxide.

c Spor-Klenz™ Ready-to-Use Cold Sterilant.

d CLIDOX-S.

e MB-10 Tablets - Quip Labs.

f Rely+On® Virkon® | Broad Spectrum Disinfectant | LANXESS.

## Conclusion

In summary, there are many considerations to take into account when setting up a GF facility. The process is much more costly than that of an SPF unit and requires specialist equipment as well as highly trained staff that are able to perform GF animal husbandry and GF lab tests. At the same time, our survey highlighted the fact that multiple options are available at all stages. Each GF unit must obey some common rules such as strict SOPs for sterile procedures, physical separation of concurrent microbial units (e.g., GF control vs repopulated group), sterilisation of diets, bedding, enrichment etc. How the procedures are designed, whether to use isolators or gnotobiotic cages, and which sterilisation methods to adopt are to be determined depending on the context. In this primer, we have outlined only some of the ways it is possible to modify procedures to maintain sterility whilst taking practical considerations into account. Careful consideration is needed as to the best set up for individual circumstances.

## Acknowledgments

We would like to thank all members of the UK Gnotobiotics Working Group for their contributions to the 2025 survey and for many useful discussions about all the issues raised in this article. We would also like to thank the National Mouse Genetic Network, in particular the Microbiome Cluster (MC_PC_21045), for useful discussions about the topic. C.F.P. is supported by the 10.13039/100016580Kennedy Trust of Rheumatology Research (KENN202115) and by the 10.13039/100010269Wellcome Trust (Investigator Award 212240/Z/18/Z awarded to Fiona Powrie). The figures were created using Biorender.com.

## Author contributions

R.B. and C.F.P.: conception and drafting and writing.

## Declaration of interests

The authors declare no competing interests.

## Supplemental information

Document S1. Figures S1and S2
