# A concise guide to setting up a germ-free unit

**DOI:** 10.1016/j.xpro.2026.104831

**Published:** 2026-09-04

**Authors:** Romuald Binet, Claire F. Pearson

## Main text

(STAR Protocols *7*, 104709; September 18, 2026)

In the originally published version of Table 8, the method for the TecCare Ultra sterilant was mistakenly described as “Silver-stabilised PAA” when it should have read “Combination: H2O2 + PAA.” This error, which occurred during manuscript preparation, has now been corrected online. The authors apologize for the oversight and regret any confusion caused.

