# Supplementary material for: A concise guide to setting up a germ-free unit
Source: STAR Protoc. 2026 Jul 16;7(3):104709. doi: 10.1016/j.xpro.2026.104709 (PMC13400397; doi:10.1016/j.xpro.2026.104709)

Supplementary Figure 1: Checklist for setting up a germ-free unit

1. Scientific needs
   - ☐ Scale of the project(s): number of animals, timescale
   - ☐ Access to collaborations or outsourcing
   - ☐ Breeding required?
   - ☐ Complexity of the experimental design

2. Available resources
   - ☐ Space available
   - ☐ Compatibility of the existing infrastructure:
     - o accessibility
     - o utilities
     - o pressure control
     - o biosecurity barrier
     - o building requirements
   - ☐ Budget available
   - ☐ Staff available
   - ☐ Contacts with equipment suppliers for quotes
   - ☐ Contacts with animal suppliers (breeders or collaborators)
   - ☐ Contacts for training, SOP and protocols

3. Breeding equipment

- Fully equipped positive pressure isolator(s)
  - ☐ Flexible film
  - ☐ Semi-rigid
  - ☐ Rigid
- Transfer/port solution
  - ☐ Rapid transfer
  - ☐ Biohazard
  - ☐ Quadro Lock
  - ☐ Swing door

4. Experimental equipment

- Gnotobiotic cages
  - ☐ Complete rack
  - ☐ Seal checker
  - ☐ Dunk tank
  - ☐ Presentation rack for autoclaving
  - ☐ Class II Biosafety Cabinet

5. Sterilisation equipment

- Autoclave
  - ☐ Validated bedding cycle
  - ☐ Validated diet cycle
  - ☐ Validated liquids cycle
  - ☐ Extra cycles as required
  - ☐ Autoclavable cylinders or drums
- ☐ Ethylene oxide steriliser
- ☐ Hydrogen peroxide vapour (VHP) system
- Chemical sterilisation
  - ☐ Peracetic acid based
  - ☐ Hydrogen peroxide based
  - ☐ Hydrogen peroxide and peracetic acid based
  - ☐ Chlorine dioxide based
  - ☐ Compressor + atomiser

6. Screening methods

- ☐ Cultures
- ☐ PCR
- ☐ Swabs vs faeces

7. Breeding consumables

- ☐ Bedding
- ☐ Enrichment
- ☐ Diets
- ☐ Water supply

8. Suppliers

- ☐ Animals
- ☐ Breeding consumables
- ☐ Equipment, including servicing
- ☐ Gamma irradiation sterilisation
- ☐ Microbiological testing

Supplementary Figure 2: Possible autoclave cycles for bedding, diet, media and fluid runs. Pulsed vacuum runs are used for bedding and diet sterilization with a temperature of 132-134°C. For liquids it is better to use a pre-vacuum process followed by active jacket cooling. To avoid liquids over boiling, temperature should be 121°C and a compressed-air cushion should be used.

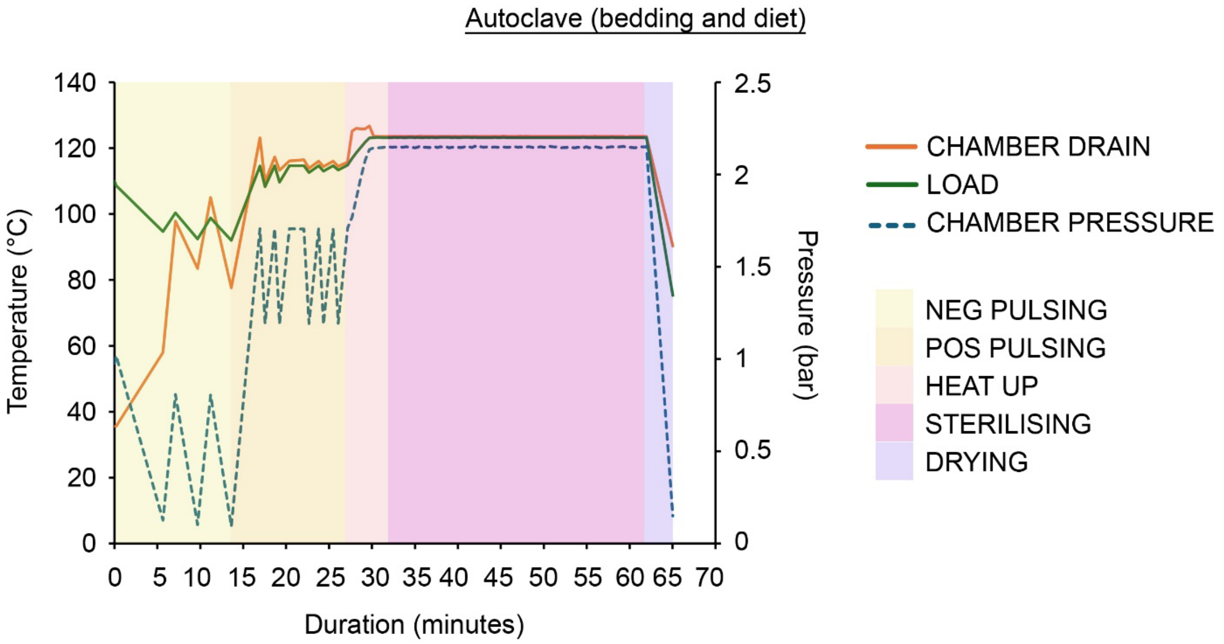

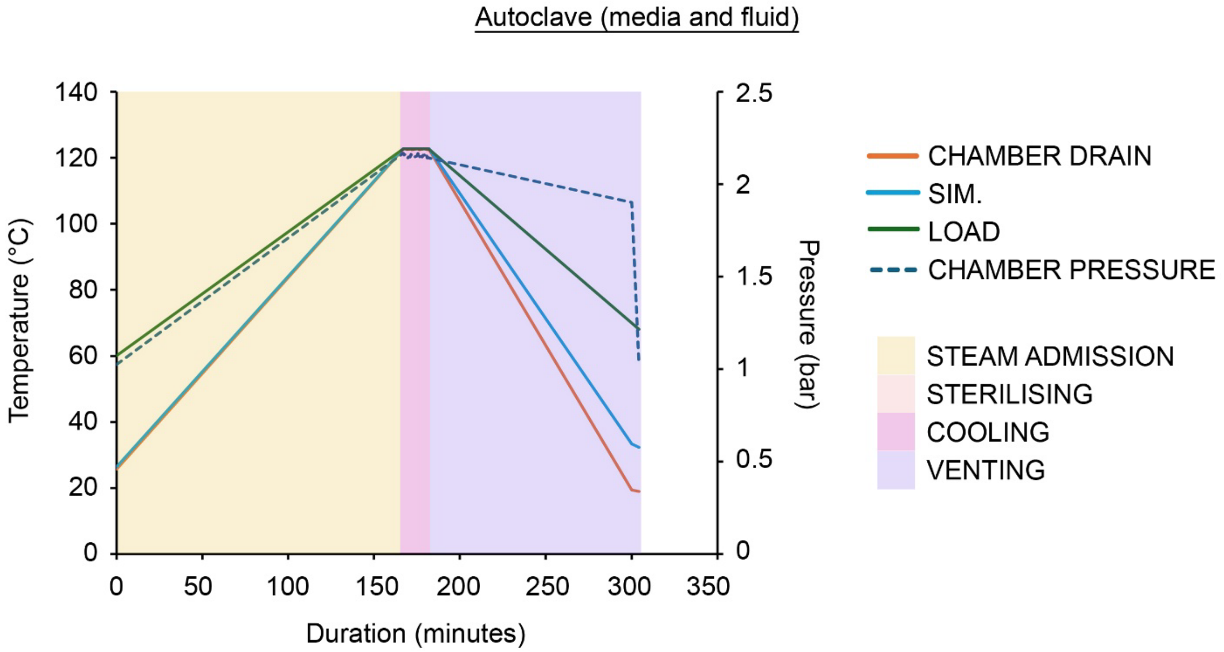

Supplement: Document S1. Figures S1and S2 [file mmc1.pdf]
